# Gene Pyramiding of Peptidase Inhibitors Enhances Plant Resistance to the Spider Mite *Tetranychus urticae*

**DOI:** 10.1371/journal.pone.0043011

**Published:** 2012-08-10

**Authors:** Maria Estrella Santamaria, Inés Cambra, Manuel Martinez, Clara Pozancos, Pablo González-Melendi, Vojislava Grbic, Pedro Castañera, Felix Ortego, Isabel Diaz

**Affiliations:** 1 Centro de Biotecnología y Genómica de Plantas (UPM-INIA). Campus Montegancedo Universidad Politécnica de Madrid, Autopista M40 (km 38), Madrid, Spain; 2 Department of Biology Western University, Ontario, Canada; 3 Dpto. Biologia Medioambiental, Centro de Investigaciones Biológicas, CSIC, Madrid, Spain; Cinvestav, Mexico

## Abstract

The two-spotted spider mite *Tetranychus urticae* is a damaging pest worldwide with a wide range of host plants and an extreme record of pesticide resistance. Recently, the complete *T. urticae* genome has been published and showed a proliferation of gene families associated with digestion and detoxification of plant secondary compounds which supports its polyphagous behaviour. To overcome spider mite adaptability a gene pyramiding approach has been developed by co-expressing two barley proteases inhibitors, the cystatin *Icy6* and the trypsin inhibitor *Itr1* genes in Arabidopsis plants by *Agrobacterium*-mediated transformation. The presence and expression of both transgenes was studied by conventional and quantitative real time RT-PCR assays and by indirect ELISA assays. The inhibitory activity of cystatin and trypsin inhibitor was *in vitro* analysed using specific substrates. Single and double transformants were used to assess the effects of spider mite infestation. Double transformed lines showed the lowest damaged leaf area in comparison to single transformants and non-transformed controls and different accumulation of H_2_O_2_ as defence response in the leaf feeding site, detected by diaminobenzidine staining. Additionally, an impact on endogenous mite cathepsin B- and L-like activities was observed after feeding on Arabidopsis lines, which correlates with a significant increase in the mortality of mites fed on transformed plants. These effects were analysed in view of the expression levels of the target mite protease genes, C1A cysteine peptidase and S1 serine peptidase, identified in the four developmental mite stages (embryo, larvae, nymphs and adults) performed using the RNA-seq information available at the BOGAS *T. urticae* database. The potential of pyramiding different classes of plant protease inhibitors to prevent plant damage caused by mites as a new tool to prevent pest resistance and to improve pest control is discussed.

## Introduction

The two-spotted spider mite *Tetranychus urticae* Koch (Acari: Techanychidae) is one of the most damaging agriculture pests worldwide. It is a polyphagous species that feeds on more than 1,100 host plants, 150 of them of economic interest, including a wide range of ornamentals, greenhouse crops and annual and perennial field cultivars [1]. The spider mite sucks the plant cell content of leaf mesophyll and in consequence chloroplasts are gradually destroyed, plant photosynthesis declines, stomata closes, and transpiration decreases leading to a reduction in crop yield. Pesticides have played a central role in spider mite control. However, because of its short generation time and high population rate, *T. urticae* has a particular ability to develop a rapid resistance to the major pesticide groups and presents a great record of pesticide resistance [2], [3]. In addition, few resistant plant cultivars are currently available and mites are not affected by Bt toxins expressed in transgenic plants [4], [5].

Recently, the complete sequence and annotation of *T. urticae* genome have been published [6]. Among other important features of spider mite genome, a large proliferation of gene families associated with digestion and detoxification of plant secondary compounds have been identified. A parallel transcriptomic analysis of spider mites feeding on different hosts has shown that expression of members of these gene families vary depending on the host, correlating with mite’s adaptability to change host environment and to its polyphagous behaviour. Mites use both extracellular and intracellular digestion, with the latter occurring in gut wall-derived epithelial cells that digest food particles that can be free floating [7], [8]. Processed food and cells pass into the posterior midgut, are subsequently compacted in the hindgut and excreted as faecal pellets [7]. The midgut is the site for synthesis and secretion of digestive enzymes and absorption of nutrients. The proteolytic digestion on mite species that feed on plants is based mostly on cysteine peptidase activities [9], [10]. This is consistent with the three-fold proliferation of cysteine peptidase gene family, mainly of C1A papain and C13 legumain classes, found in the *T. urticae* genome in comparison to other sequenced arthropod species [6]. However, serine and aspartic peptidase gene families have also been identified as important peptidases in the spider mite genome, though they are most probably involved in other physiological processes.

Peptidase activity is modulated by specific inhibitors that are grouped according to the peptidase type they inhibited [11]. Two of the most abundant plant protease inhibitors are the cystatins (family I25), which inhibit cysteine peptidases C1A and C13, and cereal trypsin/α-amylase inhibitors (family I6). Plant protease inhibitors from these two classes have been used as defence proteins against pathogens and pests due to their capability to inhibit heterologous enzymes. However, besides a defence role, they are also involved in the regulation of the plant protein turn-over required in multiple physiological processes. In barley, the complete family of cystatins, which comprises 13 genes, has been characterised and some of their members transgenically expressed in plants have conferred resistance against coleopteran, aphids and mites [10], [12], [13]. The best characterized trypsin inhibitor in barley is the *Itr1* gene encoding the BTI-CMe protein which is specifically accumulated in the developing endosperm of the grain [14]. It has been also used as a defence transgene in wheat and rice against stored grain pests such as the lepidopteran *Sitotroga cerealella* and the coleopteran *Sitophilus oryzae*, respectively [15], [16].

A number of genes with anti-mite properties have been transgenically expressed in plants to interfere with mite performance and to develop alternative strategies of plant protection. McCafferty et al. [17] reported a significant reduction in the multiplication of carmine spider mites (*Tetranychus cinnabarinus*) after feeding on papayas expressing a chitinase gene from *Manduca sexta*. The transformed papayas showed an increased tolerance both under laboratory and field trials where natural mite infestation occurred. Similarly, in papayas expressing the snowdrop GNA lectin gene affected the performance of the carmine spider mite that displayed a reduction in the feeding time and delay in egg laying [18]. Although chitinase mode of action is still not well known, it was suggested that it targeted the peritrophic membrane that encloses food in the mid and hingut, while the anti-mite activity of lectins was probably mediated by binding to chitin in the peritrophic matrix or by interacting with glycoproteins on the epithelial cells of the mite midgut [17], [18]. More recently, Carrillo et al. [10] have shown that the expression of the barley cystatin HvCPI-6 in maize impaired development and reproductive performance of *T. urticae* by inhibiting their cysteine protease activities. In contrast, experiments developed with tomato plants expressing a glucose oxidase or the soybean Kunitz inhibitor gene enhanced the *T. urticae* growth under greenhouse conditions [19].

Pyramiding (stacking) multiple defence genes in one plant has been developed as a method to prevent pest resistance and to improve pest control. Plants co-expressing a combination of enzyme inhibitors or combining them with transgenically expressed Bt toxins, lectins and thionins have enhanced plant resistance against insects when compared to plants that expressed the individual genes [20]–[24]. Based on this approach, rice lines expressing Cry1Ac and the cowpea trypsin inhibitor CpTI are awaiting approval of biosafety certificates for their release/exploitation as commercial resistant plants in China [25]. Enhancement of insecticidal activity of hydrolytic inhibitors has also been obtained by combining them with transgenically expressed lectins and thionins [22], [23], [26].

In the present study, we described a multigene approach targeted to control *T. urticae* infestation by co-expressing two barley proteases inhibitors (cystatin *Icy6* and trypsin inhibitor *Itr1* genes) in Arabidopsis plants. Single transformed lines independently expressing each transgene and double transformants have been challenged to spider mite infestation. Impact on mite survival and on endogenous mite peptidase activities have also been determined after feeding on transformed and non-transformed Arabidopsis lines. The potential of pyramiding different classes of plant protease inhibitors to prevent plant damage caused by mites is discussed.

## Results

### Molecular Characterization of Arabidopsis Plants Expressing the *Icy6* and *Itr1* Barley Genes from Barley

CMe-plants (lines 3.4 and 8.9) and CPI6-plants (lines 5.4 and 9.8) expressing trypsin and cystatin inhibitors (*Itr1* and *Icy6* genes), respectively, were used in this study [10], [27]. Additionally, double transgenic Arabidopsis plants (CPI6-CMe-plants) were generated after Agrotransformation of the single transgenic CPI-6, line 5.4 with the *Itr1* gene. Double T1 seedlings were assessed for the presence of transgene mRNAs of both genes. The T1 lines that expressed *Itr1* and *Icy6* genes were self-fertilized and progeny from the T2 generation was recovered and screened by genomic PCR to identify the presence of *Icy6* and *Itr1* genes. No phenotypic differences were observed in transformed lines in comparison to the control Columbia plants. Lines CPI6-CMe 6.4 and CPI6-CMe 8.2 were selected for further studies based on their inhibitory activity against papain and trypsin (data not shown). Independent plants of these T2 double transgenic lines exhibited the expected 321 and 534 bp bands after electrophoresis of amplified products, which were absent in the non transformed plant and in the water control (Fig. S1).

**Figure 1:**
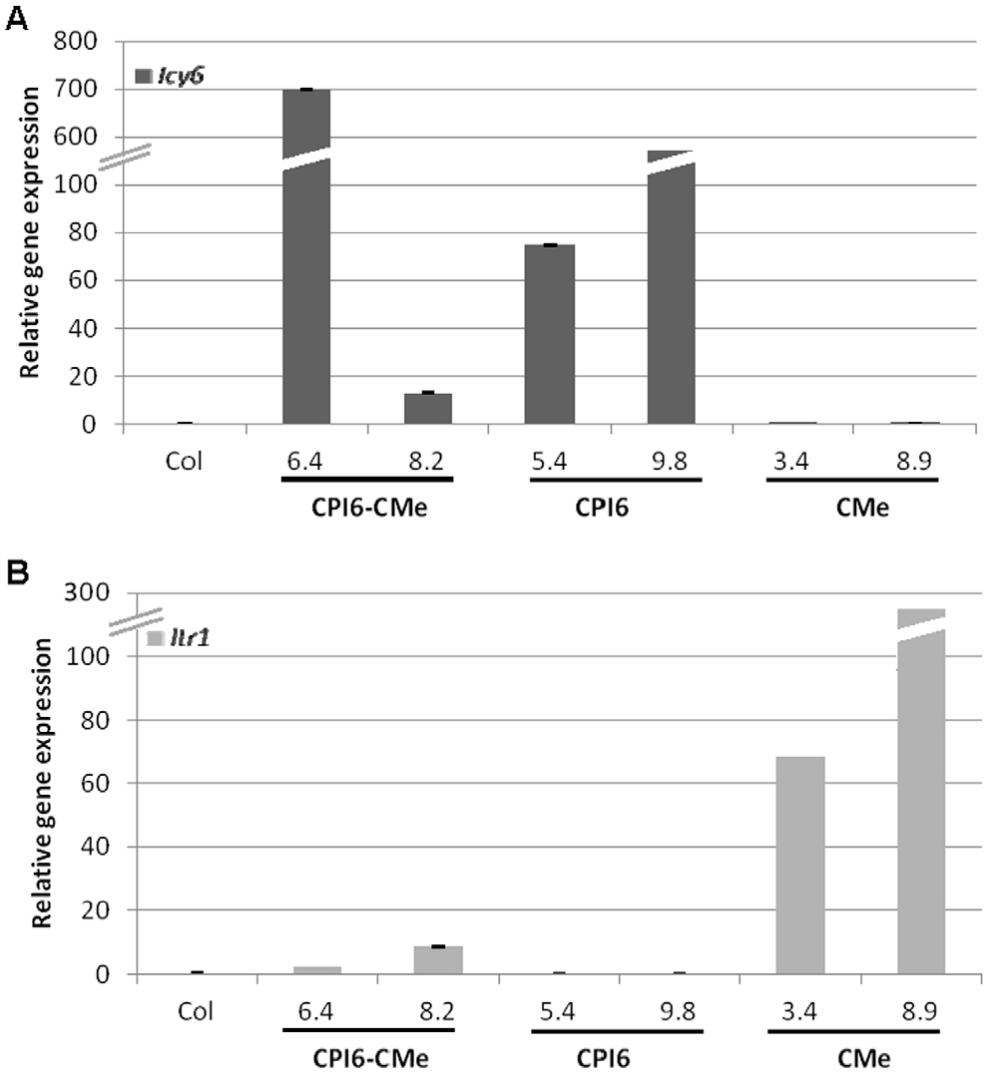
Analysis of the mRNA expression of the barley transgenes in the single (CPI6-plants, lines 5.4 and 9.8; CMe-plants, lines 3.4 and 8.9) and double (CPI6-CMe-plants, lines 6.4 and 8.2) T2 transgenic plants and non transformed control (Col), by real time quantitative PCR. A. Expression of barley *Icy6* gene. B. Expression of barley *Itr1* gene. Values expressed as the relative mRNA contents of the protease inhibitor genes were normalized to the Arabidopsis ubiquitin gene expression.

The expression of the cystatin and trypsin inhibitor genes in Arabidopsis transformed and non-transformed control (Col) plants was analysed by real-time quantitative PCR (qRT-PCR) using specific primers and the content of cystatin/trypsin inhibitor mRNAs was normalized to Arabidopsis ubiquitin transcript levels. Strong differences in the expression of transgenes among different transgenic lines were observed (Fig. 1). While cystatin messengers were highly expressed in the CPI6-CMe 6.4 line, *Icy6* mRNA expression levels were much lower in the CPI6-CMe 8.2, and expression of the trypsin inhibitor *Itr1* gene was comparatively lower in both double transgenic lines. In addition, qRT-PCR analyses were performed in single transgenic lines independently expressing the *Itr1* or the *Icy6* genes, selected for this study. Again, strong differences on the mRNA expression levels among transgenic lines were observed for CPI6-plants (lines 5.4 and 9.8) and CMe-plants (lines 3.4 and 8.9). As expected, no *Icy6* transcripts were detected in the RNA isolated from CMe-plants (lines 3.4 and 8.9) neither was *Itr1* mRNA was found in the CPI6-plants (lines 5.4 and 9.8). Similarly, *Icy6* and *Itr1* messengers did not appear in the non-transformed Col plants.

**Figure 2:**
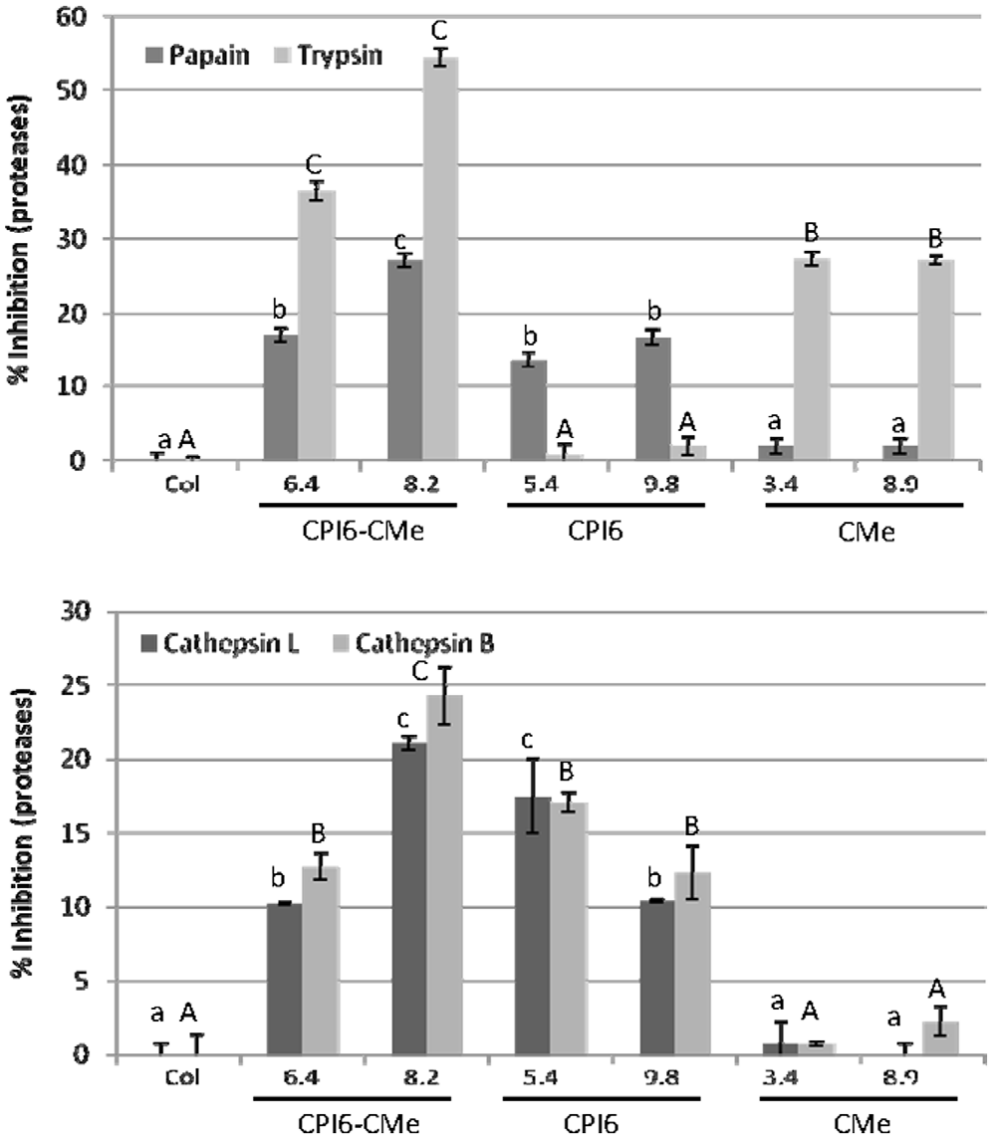
Inhibitory activity of protein extracts from transformed lines and non-transformed control against commercial proteases and *T. urticae* extracts. **A.** Inhibitory activity of commercial papain and trypsin using Z-FR-AMC and ZLA-AMC as substrates. **B.** Inhibitory activity of spider mite protein extracts using Z-RR-AMC and Z-FR-AMC as substrates. Data are mean ± SE of triplicate measurements of each sample. Different letters indicate significant differences (P<0.05, Student-Newman-Keuls test).

Transformed and control Arabidopsis lines were also used to analyse the presence of the cystatin protein in leaf extracts by indirect ELISA (iELISA) assays to analyse variations in protein and mRNA expression levels. As shown in Fig. S2, the barley cystatin protein immobilized on a plastic substrate was detected with the anti-cystatin peptide antibody and subsequently quantified by a secondary alkaline phosphatase-conjugated antibody. Protein was accumulated at higher concentration in plants expressing the *Icy6* as a single transgene (CPI-6) than in plants expressing both inhibitors (CPI6-CMe). Additionally, *in vitro* inhibitory activity was performed with protein extracts derived from all Arabidopsis lines against commercial papain, trypsin and *T. urticae* extracts. Results, quantified by the decreased amount of substrates hydrolyzed by the papain and trypsin, were expressed as percentage of inhibitory enzyme activity (Fig. 2A). Transgenic lines over-expressing the *Icy6* gene (CPI6-CMe-plants: lines 6.4 and 8.2 and CPI6-plants: lines 5.4 and 9.8) showed significant inhibitory activity against papain over the values obtained with the protein extracts from the non-transformed control plants Similarly, transformed CPI6-CMe- and CMe-lines presented a greater ability to inhibit commercial trypsin than did extracts from the control plants. Interestingly, the double transformed lines showed higher inhibitory capability against both commercial proteases. As expected, no papain inhibition was detected in CMe-plants neither trypsin inhibition was observed in the CPI6-plants.

To know if these inhibitors affect protease activity in mite extracts, Arabidopsis protein were tested using Z-FR-AMC and Z-RR-AMC substrates susceptible to be hydrolysed by cathepsin L- and B-like activities, respectively. As is shown in Fig. 2B, single or double transformed lines over-expressing the *Icy6* gene showed an inhibitory capability against cathepsin B- and L-like activities slightly lower to that obtained against commercial papain, which was not detected in control plants nor in the transformed CMe-plants. Trypsin inhibition could not be determined on mite protein samples because the activity was too low to be accurately measured (data not shown), which was congruent with the absence of trypsin activity in mite extracts reported by Carrillo et al. [10].

### Spider Mite Feeding Damage on Arabidopsis Lines

**Figure 3:**
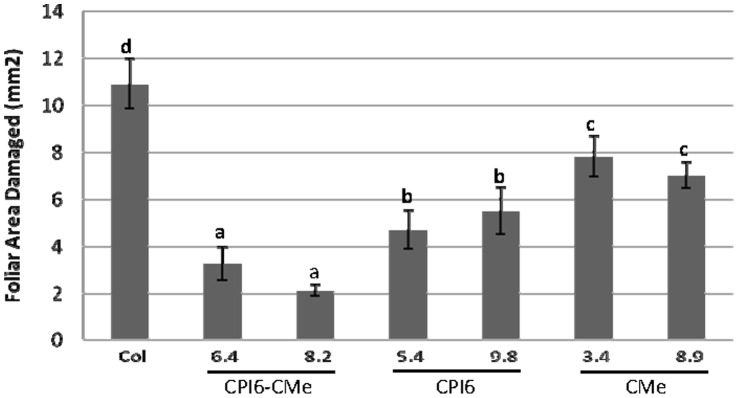
Leaf damage on single and double Arabidopsis transformed lines and non-transformed control 4 days after *T. urticae* infestation. Data are mean ± SE of twelve measurements divided in two experimental blocks. Different letters indicate significant differences (P<0.05, Student-Newman-Keuls test).

To investigate the effect produced by *T. urticae* on transformed and non-transformed lines, leaf damage was quantified after 4 days of mite feeding on entire Arabidopsis plants. All T2 transgenic lines, independently of single or double transgene integration, showed significant less damaged leaf area than leaves from non-transformed control (Fig. 3). Interestingly, double transgenic lines CPI6-CMe 6.4 and 8.2 showed the highest resistance to mite damage: 3.2 and 2.1 mm^2^ of damaged leaf area, respectively, in comparison to the 10.91 mm^2^ of damage area detected in the control plant. Lines over-expressing the cystatin gene resulted significantly more resistant (about 4–5 mm^2^ of total leaf damage) than lines over-expressing the trypsin inhibitor (about 7 mm^2^).

Upon feeding, mites induced the accumulation of H_2_O_2_ at the leaf-feeding site which can be detected by the brown colour of the oxidized diaminobenzidine (DAB) used as substrate in the histochemical assays. To further corroborate the leaf damage results, DAB-H_2_O_2_ reaction product was determined in the transformed and no-transformed lines after mite feeding. Control plants stained more intensely than any of the transformed lines (Fig. S3). Double transgenic leaves resulted more resistant to spider mites, showed less damage leaf area and in consequence should produce less H_2_O_2_. No H_2_O_2_ was detected in non-infested Arabidopsis leaves.

### Effects of Arabidopsis Transgenic Plants on Mites

**Figure 4:**
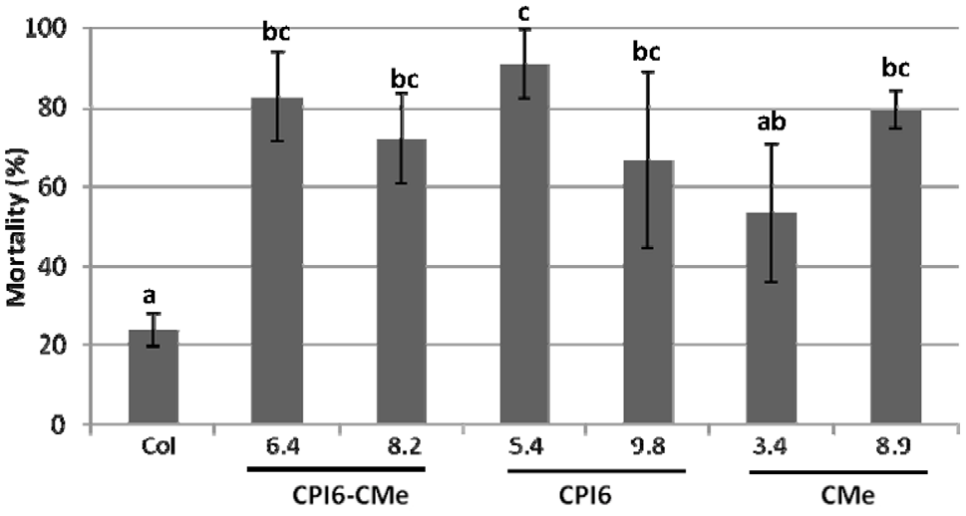
Effects of barley protease inhibitors expressed in single and double transformed plants and in non-transformed controls on *T. urticae* mortality, 10 days after infestation with neonate larvae. Different letters indicate significant differences (P<0.05, Student-Newman-Keuls test).

T2 plants of the transformed and non-transformed lines were used to analyse the transgene effect on *T. urticae* survival. As it is shown in Fig. 4, mite mortality quantified after 10 days of infestation reached values between 50 and 90% when mites fed on transformed lines compared to the 23% on non-transformed plants. Developing time from newborn larvae to nymph lasted 6.7±3 days for mites fed on control plants, whereas ranged from 7.4 to 9.7 when fed on transformed lines (Table S1). However, these differences were only statistically significant between CMe 8.9 and the control group, because of the high variability due to the low rate of mite survival on all transgenic lines.

**Figure 5:**
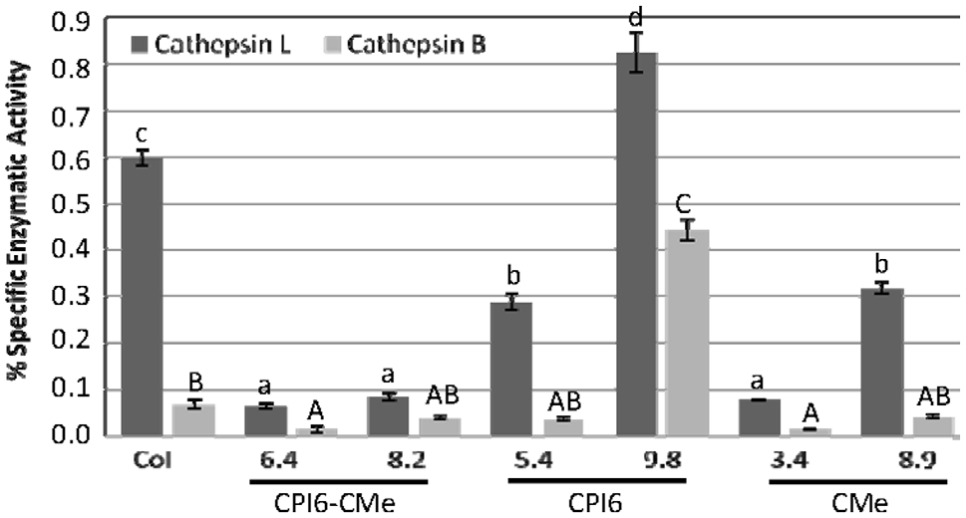
Specific proteolytic activities of cathepsin B- and L-like in *T. urticae* after feeding for 7 days on single and double transformed lines and non-transformed control using specific substrates. Data, expressed as nmoles/min/mg, are mean ± SE of triplicate measurements of each sample. Different letters indicate significant differences (P<0.05, Student-Newman-Keuls test).

Biochemical analyses were carried out on mites after feeding on transformed and control plants for 7 days. The specific activity of cathepsin L-like peptidase detected in mite protein extracts was higher than the cathepsin B-like specific activity when mites were reared on the control Arabidopsis plants. After feeding on transgenic lines, these proteolytic activities were significantly reduced when compared to those of mites fed on non-transformed control (Fig. 5). Exceptionally, a significant increase in the specific activity of both cathepsin L- and B-like was observed in extracts from mites reared on the transgenic line CPI6 9.8. Specific trypsin activity was also tested but no activity was detected (data not shown).

### Expression Profiling of C1A Cysteine Peptidases and S1 Serine Peptidases in *T. urticae*

**Figure 6:**
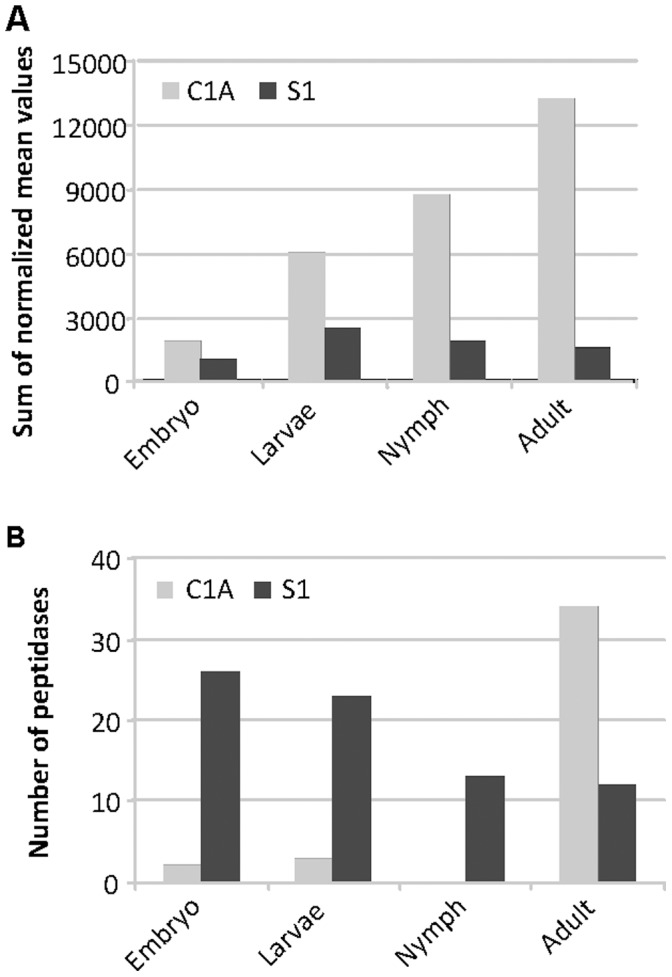
Expression profiling of C1A cysteine and S1 serine peptidase families in *T. urticae*. **A.** Sum of the normalized expression values for all C1A and S1 family members in each developmental stage analyzed. **B.** Number of genes for the C1A cysteine and S1 serine peptidase groups assigned to the developmental stage (embryo, larvae, nymph and adult) in which their highest expression was detected.

The expression levels of potential mite targets for cystatin *Icy6* and trypsin inhibitor *Itr1* genes were analyzed by an *in silico* assays using the RNA-seq information available at the BOGAS *T. urticae* database [28]. Transcriptomic information was available for the 57 genes of C1A cysteine peptidase and for 125 genes of S1 serine peptidase. Figure 6A shows the sum of the normalized mean values of gene expression for both families of enzymes at different mite developmental stages. Genes with an expression value lower than five (14 and 51 genes of C1A and S1, respectively) were discarded from further analysis. Figure 6B shows the distribution of numbers of C1A cysteine and S1 serine peptidases that reach maximum expression over mite developmental stages. Genes belonging to the C1A family are reaching maximum expression at later stages, mainly in the adult phase, while most genes of the S1 family were expressed in similar pattern throughout the mite life cycle, although an important set of genes had their maximum expression values at the embryo and larvae stages.

## Discussion

The two-spotted spider mite, *T. urticae,* is one of the most striking examples of polyphagy among herbivores. The recent sequencing and annotation of the spider mite genome has discovered a high number of detoxification gene families associated with plant feeding and a proliferation of peptidase genes putatively involved in digestion that may support the expression of the spider host range [6]. The abundance of cysteine peptidase genes, particularly C1A papain, is consistent with its proteolytic digestion based mostly on cysteine peptidase activity [9], [29]. Previous findings on the characterization of protease activities of *T. urticae* corroborated the presence of these enzymes and have shown their susceptibility as targets of cystatins. Additionally, *in vitro* inhibitory assays have demonstrated that the HvCPI-6 cystatin (gene *Icy6*) purified as recombinant protein was the strongest inhibitor against spider mite cathepsin B- and L-like activities [10]. Besides the proliferation of cysteine proteases found in the spider mite genome, a large serine-protease gene family was also identified [6]. Thus, serine proteases including trypsin- and chymotrypsin-like proteases have to be essential in the spider mite physiology although they are probably not directly involved in the hydrolytic digestion of dietary proteins. These genomic features show the presence of putative mite targets for *Itr1* and *Icy6* transgenes encoding trypsin and cystatin inhibitors from barley. Transformed plants containing either one or both of these transgenes were used to analyse the putative acaricide effects on *T. urticae* and to test their ability to protect plants against the spider mite infestation.

The accumulation of *Itr1* or/and *Icy1* transcripts detected in Arabidopsis lines was associated with the inhibition of commercial papain and trypsin detected by *in vitro* assays using plant extracts. However, Arabidopsis lines with high mRNA levels do not always correspond to the lines with the greater protein accumulation or the maximum inhibition activity. This may be due to differences on the gene copy number inserted into the plant genome or on the protein expression levels, as is exemplified by the cystatin accumulation detected by iELISA assays. These differences on mRNA and protein expression are a general characteristic previously described in transgenic plants [13], [17]. Feeding trials conducted with the spider mite resulted in a significant reduction of leaf damage and an increase in mite mortality, observed in all the transformed lines analysed in comparison to non-transformed control. In this context, the most interesting observation was that the double transgenic lines, and in particular the line 8.2, had the greatest inhibitory properties not only against commercial proteases but also against cathepsin L- and B-like cysteine proteases from *T. urticae* extracts. These results are strongly correlated with the reduction in the leaf damage detected in the Arabidopsis lines expressing both inhibitors after 4 days of mite feeding. Moreover, the retardation tendency in the larvae development, after feeding on transformed Arabidopsis leaves, particularly detected in lines expressing the trypsin inhibitor, corroborate the role of the serine proteases in the spider mite growth.

The effect of the two transgenes may be additive or synergistic depending on the gene combination, although here it is difficult to ascertain the transgene relationship since the trypsin inhibitor protein levels have not been determined. Nevertheless, it is clearly shown that double transgenic lines presented a significant reduction in leaf damage either quantified as total chlorotic area or detected by DAB staining in comparison to the independent single transformants. These results were clarified by an *in silico* analysis of transcriptome expression that was performed using the RNA-seq information available at the BOGAS *T. urticae* database, where most genes for C1A cysteine and S1 serine peptidase genes have transcriptomic information. The sum of all normalized C1A peptidase genes in the four developmental mite stages (embryo, larvae, nymphs and adults) resulted much higher than the total expressed S1 serine peptidase genes, which did not show a clear specific developmental pattern of expression. Furthermore, the most expressed genes belonging to the C1A peptidase group were highly abundant in the last stages of mite development, mainly in the adult phase, while S1 serine peptidase genes did not show a clear specific developmental pattern of expression. These results are in agreement with a primarily digestive role of cysteine protease in mites [6], [10]. In contrast, the presence of highly expressed S1 serine peptidase genes in embryo and larvae suggest that they may have other putative roles, probably associated with the regulation of growth and development. These physiological processes can potentially be targeted if the protease inhibitors may get access through the mite gut to endogenous targets, as have been already reported in insects [30], [31].

The characterization of specific cathepsin B- and L-like protease activities of *T. urticae* after feeding on transgenic lines substantiated the impact of the barley cystatin on mite target peptidases. The clear decrease on both cathepsin-like specific activities confirmed the potential for direct interference of HvCPI-6 cystatin on *T. urticae* digestion. The inhibition of proteolysis through PIs may decrease access to essential amino acids and consequently protein functions can be impaired disrupting crucial physiological events of *T. urticae* such as nutrition, redox status, development, reproductive performance, etc., which finally increase their mortality, as it is demonstrated in this work. However, mites possess a remarkable ability to adapt their metabolism to the dietary material ingested and can show different compensatory responses to host plants expressing distinct sets of defence proteins [2], [6], [19]. Arthropods can also employ a battery of tactics to avoid the effects of plant defences (PIs in this case) by compensating or by adjusting their proteases through modulation of transcripts and/pr protein products. In addition, they can even modify the efficiency of posttranslational features, particularly under inhibitor challenges, which directly correlates with varied proteolytic activity of different protein isoforms [32], 33. The over-expression of target proteases is a common strategy to counteract the inhibitory activity [34], [35], which could explain the increases of cathepsin L- and B-like specific activities observed on line 9.8 of the transformed CPI6-plants. Similarly, the induction of novel insensitive proteases and the physiological complementation by non-target proteases of other mechanistic classes have also been described as pest adaptive processes [12], [35], [36]. It could explain the non-expected reduction of cathepsin L-like specific activity found in *T. urticae* reared with single transformed lines 3.4 and 8.9, expressing the CMe trypsin inhibitor. Besides, we can not discard the possibility that some serine proteases may be involved in the cysteine protease processing needed for this peptidase to become active [37]. In this scenario, the transgene pyramiding targeting different physiological process in mites will make more difficult to the spider mite to overcome defences and to create counter defences.

In conclusion, pyramiding two barley protease inhibitor genes in Arabidopsis genome have resulted more effective to enhance *T. urticae* control than a single transgene expression by conferring leaf protection against spider mite damage. An additional advantage is that this approach may prevent the development of spider mite adaptive mechanisms directed to overcome the expression of single miticidal proteins and in consequence, makes it more difficult to overcome plant resistance. Our approach also highlights the benefits of the access to genomic and other ‘omic information for the identification of candidate target sites that may have a positive impact in pest control.

## Materials and Methods

### Plant Material

Arabidopsis plants independently expressing the barley *Icy6* gene encoding the HvCPI-6 cystatin without its signal peptide (35S-*Icy6*-plant, in this article CPI6-plants) and the barley *Itr1* gene encoding the BTI-CMe trypsin inhibitor (35S-*Itr1*-plants, in this article CMe-plants), previously described by Carrillo et al. [13], [27], were used in this study. Additionally, double transgenic plants (CPI6-CMe-plants) were generated by the *Agrobacterium*-mediated floral dip method [38]. The construct containing the *Itr1* gene under the CaMV35S promoter and the *NptII* selective gene previously used by Carrillo et al. [13] was integrated into the genome of the transgenic line 5.4 of CPI6-plants. Seeds from double transformed plants were harvested and plated on MS-medium containing 50 µg/ml Kanamycin and the resultant seedlings were transplanted to soil and allowed to set seeds. T2 seeds were harvested and tested for the presence of the two transgenes by PCR before further characterization and mite bioassays.

*Arabidopsis thaliana* transgenic and non-transformed Col plants were grown under control conditions (23°C, 70% relative humidity and a 16 h/8 h day/night photoperiod).

### Spider Mites

A colony of *T. urticae,* London strain (Acari: Tetranychidae), provided by Dr. Miodrag Grbic (UWO, Canada), was reared on beans (*Phaseolus vulgaris*) and maintained on growth chambers (Sanyo MLR-350-H, Sanyo, Japan) at 23°C±1°C, >70% relative humidity and a 16 h/8 h day/night photoperiod.

### Nucleic Acid Analysis

Total DNA was isolated from control and T2 transgenic Arabidopsis lines (CPI6-CMe: lines 6.4 and 8.2; CPI6: lines 5.4 and 9.8; CMe: lines 3.4 and 8.9) essentially as described by Sambrook and Russell [39] and tested for the presence of cystatin and/or serine protease inhibitors genes by PCR using the following primers:

35S-F: 5′-CACTATCCTTCGCAAGACC-3′,

CPI6-R: 5′-CGAGGTACCTTAGCCGCCGGCAGCCGG-3′ and

CMe-R: 5′-CGAGGTACCTTACAAGACCAC-3′.

The PCR conditions were 40 cycles with 30 sec at 92°C, 30 sec at 55°C and 1.30 min at 72°C. The reaction products were separated on 1% agarose electrophoresis gels.

For quantitative real time PCR (qRT-PCR) studies, Arabidopsis rosettes from transformed and control lines were collected, frozen into liquid N_2_ and stored at −80°C until used for RNA isolation. Total RNA was extracted by the phenol/chloroform method, followed by precipitation with 8 M LiCl [40]. cDNAs were synthesized from 2 µg of RNA using the Revert Aid™ H Minus First Strand cDNA Synthesis Kit (Fermentas) following manufacturer’s instructions. The qRT-PCR conditions were 40 cycles with 15 sec at 95°C, 1 min at 55°C and 5 sec at 65°C. FastStart Universal SYBR Green Master (Rox) (Roche) using a total volume of 20 µl. PCR reactions were performed in multiplate PCR plates (BioRad). The reactions were carried out in a C1000™ thermal cycler with CFX96™ optical reaction module (BioRad) and results were analysed using CFX Manager Software 2.0 (BioRad). For negative controls, 1 µl of water was used instead of cDNA, as well as an RNA sample without reverse transcription (no-RT). Primer efficiency was tested using a standard curve for each gene. After amplification, a melting curve analysis was performed to verify gene specificity. The absence of genomic DNA was confirmed by the no-RT control. Reactions were performed for triplicate samples. Gene expression values were referred as relative expression or 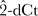. After testing that ubiquitin gene was not differentially expressed, values were normalized to Arabidopsis ubiquitin mRNA levels. The primers used for qRT-PCR amplification were:

qRT-CPI6-F: 5′-GCGGACGGCTCCGGCAAGAG-3′;

qRT-CPI6-R: 5′-AAGGACGTGAGCTTGCGGGT-3′;

qRT-CMe-F: 5′- TCCTCACCTCGGACATGAAGA-3′;

qRT-CMe-R: 5′- CCCTGCCAAGTTACTACCCCTT-3′;

qRT-Ubi-F: 5′-GAGCCTTACAACGCTACTCTGTCTGTC-3′;

qRT-Ubi-R: 5′-ACACCAGACATAGTAGCAGAAATCAAG-3′.

### Protein Detection by Indirect ELISA

Plant protein extracts were prepared from frozen transgenic and control Arabidopsis leaves. Samples were ground and resuspended in a sodium carbonate-bicarbonate extraction buffer pH 9.6, containing 15 mM sodium carbonate, 28.4 mM sodium bicarbonate and 1% polyvynilpyrrolidone-40 (PVP-40) following Hnasko et al. [41]. After quantification of protein concentration as described Bradford [38], 100 µg of total protein were applied to flat-bottom 96-well plates and incubated for 1 h at 37°C. Wells were washed with phosphate-buffered saline with 0.05% Tween-20 (PBST). 100 µl of primary anti-cystatin antibody at the optimal dilution 1∶200 (v/v) in PBST and 2% (w/v) PVP-40 were incubated overnight at 4°C. The HvCPI-6 cystatin antibody was a specific polyclonal antibody against 16 amino acids (G36 to L51 from the initial Met) of the HvCPI-6 protein, produced in rabbits by Pineda Antibody Services (Berlin, Germany). Plates were washed with PBST and incubated with 100 µl of secondary Alkaline Phosphatase-conjugated antibody (Chemicon Internationals, USA) diluted to 1∶1500 (v/v) in PBST and 2% (w/v) PVP-40 for 1 h at room temperature. One mg/ml of phosphatase substrate (Sigma Aldrich, USA) was added and absorbance was measured using a 405 nm wavelength filter. Triplicate assays were performed for determination of each value and the average was calculated. Blanks were used to account for the spontaneous breakdown of substrates. Data were normalized to the Columbia non-transformed control.

### Inhibitory Activity of Protein Extracts of Transgenic and Control Arabidopsis Lines Against Papain and Trypsin

Total protein extracts from the selected T2 transgenic and non-transformed Arabidopsis rosettes were ground and resuspended in 0.15 M NaCl sodium phosphate pH 6.0, 2 mM EDTA for 1 hour at 4°C and treated as described in Alvarez-Alfageme et al. [12]. Total protein content was determined according to the method of Bradford [42].

Inhibitory activity of plant protein extracts was *in vitro* tested against commercial papain (EC 3.4.22.2) and trypsin (EC 3.4.21.4) from Sigma. The Z-FR-AMC (N-carbobenzoxyloxy-Phe-Arg-7-amido-4-methylcoumarin) substrate was used for papain, trypsin-like activity was assayed using ZLA-AMC (z-L-Arg-7-amido-4-methyl coumarin). Basically, 20 µg of protein extracts were preincubated for 10 min with 100 ng of papain in a buffer 100 mM sodium phosphate pH 6.0, L-cysteine, 10 mM EDTA, and 0.01% (v/v) Brij35 or with 100 ng of trypsin in the buffer Tris-HCl 0.1 M, pH 7.5. Subsequently, substrates were added at a final concentration of 0.2 mM and incubated 1 h at 28°C.

Fluorescence was measured using an excitation filter of 365 nm and an emission filter of 465 nm (Tecan GeniusPro). The system was calibrated with known amounts of AMC hydrolysis product in a standard reaction mixture. Results were expressed as a percentage of protease activity relative to that in the absence of the inhibitor. All assays were carried out in triplicate and blanks were used to account for spontaneous breakdown of substrates.

### Inhibitory Activities of Protein Extracts of Transgenic and Control Arabidopsis Lines Against Mites

Spider mites reared on control Arabidopsis plants were homogenized in 0.15 M NaCl (600 mg/ml), centrifuged at 10,000 rpm for 5 min and the supernatants pooled to obtain soluble protein extracts for enzymatic activity assays. Total protein content was determined according to the method of Bradford [42]. Inhibitory activity of plant protein extracts from control and transgenic lines prepared as indicated above was *in vitro* tested using 10**µg of mite protein extracts. Inhibitory assays were performed using Z-FR-AMC and Z-RR-AMC (N-carbobenzoxy-loxy-Arg-Arg-7-amido-4-methylcoumarin) substrates for cathepsin L- and B-like activities, respectively, and the Z-LA-AMC substrate for trypsin assays, following buffers and conditions mentioned above.

### Leaf Damage Quantification on Arabidopsis Plants after Mite Feeding Assays

Damage quantification analysis were done on Arabidopsis entire T2 plants from selected transgenic lines (CPI6-CMe: lines 6.4 and 8.2; CPI6: lines 5.4 and 9.8; CMe: lines 3.5 and 8.9) and from the non-transformed control. Three week old plants were infected with 20 adults of *T. urticae* per plant. After 4 days of infestation, the leaf damage was assessed by scanning the entire rosette using a hp scanjet (HP Scanjet 5590 Digital Flatbed Scanner series), according to Navarro et al. [43]. Leaf damage was calculated in mm^2^ using Adobe Photoshop CS software. Twelve measurements divided in two experimental blocks were used for each genotype.

The detection of H_2_O_2_ accumulation in response to mite damage was analysed using 3,3-diaminobenzidine tetrachloride hydrate (DAB) substrate which produces a brown precipitate after oxidation in the presence of H_2_O_2_ [44]. The staining procedure used was reported by Rodríguez-Herva et al. [45] and observed under a Zeiss Axiophot microscope.

### Mite Bioassays on Arabidopsis Plants Expressing the Barley Cystatin and/or Serine Protease Inhibitors

Mite bioassays were conducted on Arabidopsis entire detached leaves derived from T2 plants of the selected transgenic lines (CPI6-CMe: lines 6.4 and 8.2; CPI6: lines 5.4 and 9.8; CMe: lines 3.5 and 8.9) and from the plant control. Entire leaves were placed onto wet cotton, surrounded by wet filter paper to avoid mite escapes in confined Petri dishes. Samples were maintained under controlled conditions at 23°C±1°C, >70% relative humidity and a 16 h/8 h day/night photoperiod. Fifteen neonate larvae (<24 h) of *T. urticae* were placed on each leaf and mortality recorded after 10 days. Six replicates (from different plants) of every transgenic line and non-transformed control were done.

### Protease Activity of *T. urticae* Protein Extracts after Feeding on Arabidopsis Lines

Protease activity of *T. urticae* was analysed after 7 days of feeding on control and transgenic Arabidopsis lines. Mites were collected and stored frozen (−20°C) until needed. Mites were homogenized in 0.15 M NaCl (600 mg/ml), centrifuged at 10,000 rpm for 5 min and the supernatants pooled to obtain soluble protein extracts for enzymatic activity assays. Total protein content was determined according to the method of Bradford [42].

The standard assay volume was 100 µl, using 5 µg of mite protein extract and the corresponding substrate added to a final concentration of 0.2 mM. Cathepsin B- and L-like and trypsin activities were assayed as described above using Z-RR-AMC, Z-FR-AMC and ZLA-AMC substrates, respectively. The reaction was incubated 2 hours at 28°C and emitted fluorescence measured and calibrated as indicated above. Specific enzymatic activity was calculated as nmoles of substrate hydrolyzed/min/mg protein. All assays were carried out in triplicate and blanks were used to account for spontaneous breakdown of substrates.

### Statistic Analysis

Differences in inhibitory activity, leaf damage, mortality, development and proteolytic activities were compared by on-way ANOVA, followed by Student-Newman-Keuls multiple comparison tests. Percentage data (inhibitory activity and mortality) were transformed using arcsin square root transformation to normalize distributions and stabilize the variance before statistical analysis.

### In Silico Transcriptome Expression

The transcriptomic information available at the BOGAS T. urticae website (Bogas;http://bioinformaticspsbugentbe/webtools/bogas/overview/Tetur *]*. ) was used to the developmental expression analyses. The protocol to normalized read counts of RNA-seq Illumina reads has been previously described [6]. T. urticae C1A genes were previously reported in [6]. 120 spider mite S1 genes were automatically selected from the GO annotation of the transcriptome. When their S1 features were manually checked 114 of these genes belonged to the S1 family. Eleven additional S1 genes with transcriptomic data were obtained by recurrent BLAST searches in the T. urticae database using spider mite S1 sequences.

## Supporting Information

Figure S1 **PCR analysis of T2 Arabidopsis double transformed with** ***Icy6*** **and** ***Itr1*** **barley genes, encoding the cystatin HvCPI-6 (CPI6) and the trypsin inhibitor (CMe), respectively.** Genomic PCR was performed using the forward and reverse primers derived from the CaMV35S promoter and the 3′region of the *Icy6* or *Itr1* genes, respectively. Plants are: double transgenic CPI6-CMe plants (lines 6.4 and 8.2) and non transformed control (Col). H_2_0: water control. M: molecular size marker.
(PDF)

Figure S2 **Detection of HvCPI-6 barley cystatin in transgenic Arabidopsis lines by iELISA assays.** Leaf protein extracts (100 µg) were immobilized by adsorption into 96-well microplates and HvCPI-6 protein detected with the cystatin peptide antibody and subsequently quantified by a secondary alkaline phosphatase-conjugated antibody. Data are mean ± SE of triplicate measurements of each protein extract sample. Different letters indicate significant differences (P<0.05, Student-Newman-Keuls test).
(PDF)

Figure S3 **Histochemical detection of reactive oxygen species (ROS) in leaves of control uninfected plants (a) and leaves in response to 24 hours spider mite feeding: b) control Arabidopsis; c) CMe 3.4 line; d) CPI6 6.4 line and e) CPI6-CMe 8.4 line.**
(PDF)

Table S1 **Effects of the transgenic Arabidopsis lines on** ***T. urticae*** **development after feeding assay.**
(PDF)
