# Supplementary figures and images for: Gene Pyramiding of Peptidase Inhibitors Enhances Plant Resistance to the Spider Mite Tetranychus urticae
Source: PLoS One. 2012 Aug 10;7(8):e43011. doi: 10.1371/journal.pone.0043011 (PMC3416837; doi:10.1371/journal.pone.0043011)

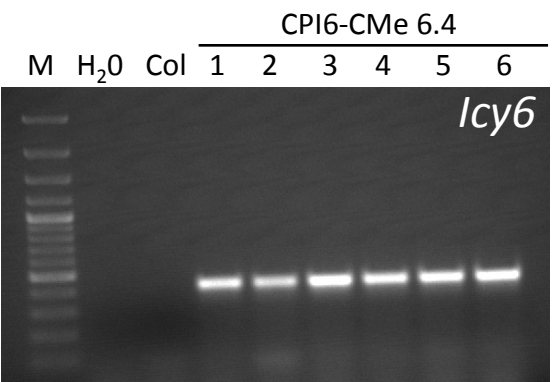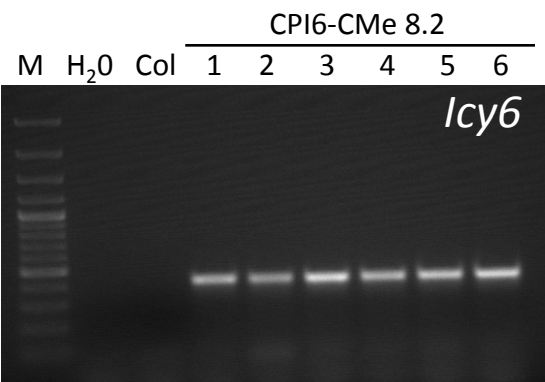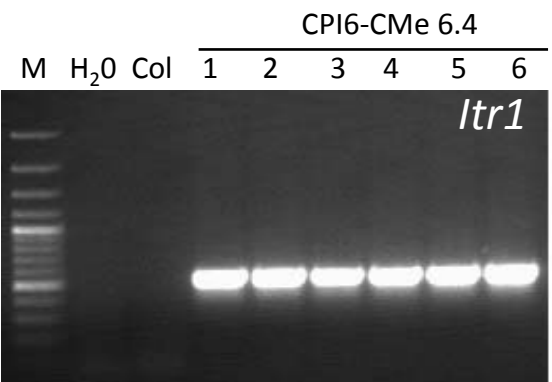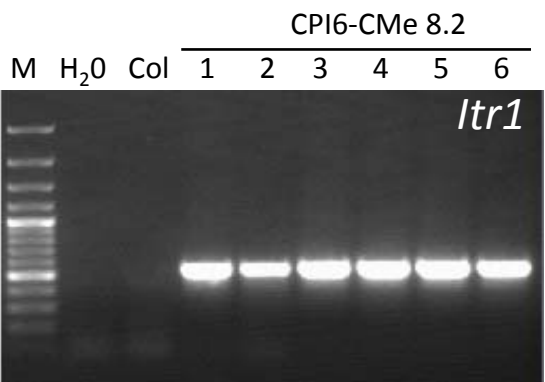

Figure S1

Supplement: Figure S1 — PCR analysis of T2 Arabidopsis double transformed with Icy6 and Itr1 barley genes, encoding the cystatin HvCPI-6 (CPI6) and the trypsin inhibitor (CMe), respectively. Genomic PCR was performed using the forward and reverse primers derived from the CaMV35S promoter and the 3′region of the Icy6 or Itr1 genes, respectively. Plants are: double transgenic CPI6-CMe plants (lines 6.4 and 8.2) and non transformed control (Col). H20: water control. M: molecular size marker. (PDF) [file pone.0043011.s001.pdf]

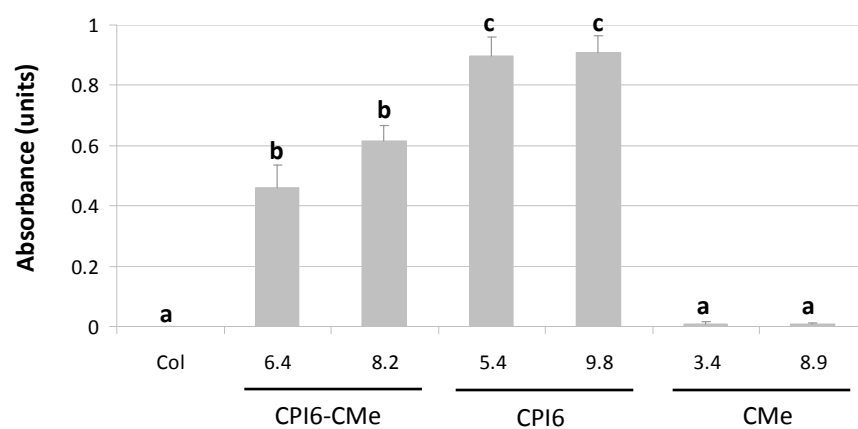

Figure S2

Supplement: Figure S2 — Detection of HvCPI-6 barley cystatin in transgenic Arabidopsis lines by iELISA assays. Leaf protein extracts (100 µg) were immobilized by adsorption into 96-well microplates and HvCPI-6 protein detected with the cystatin peptide antibody and subsequently quantified by a secondary alkaline phosphatase-conjugated antibody. Data are mean ± SE of triplicate measurements of each protein extract sample. Different letters indicate significant differences (P<0.05, Student-Newman-Keuls test). (PDF) [file pone.0043011.s002.pdf]

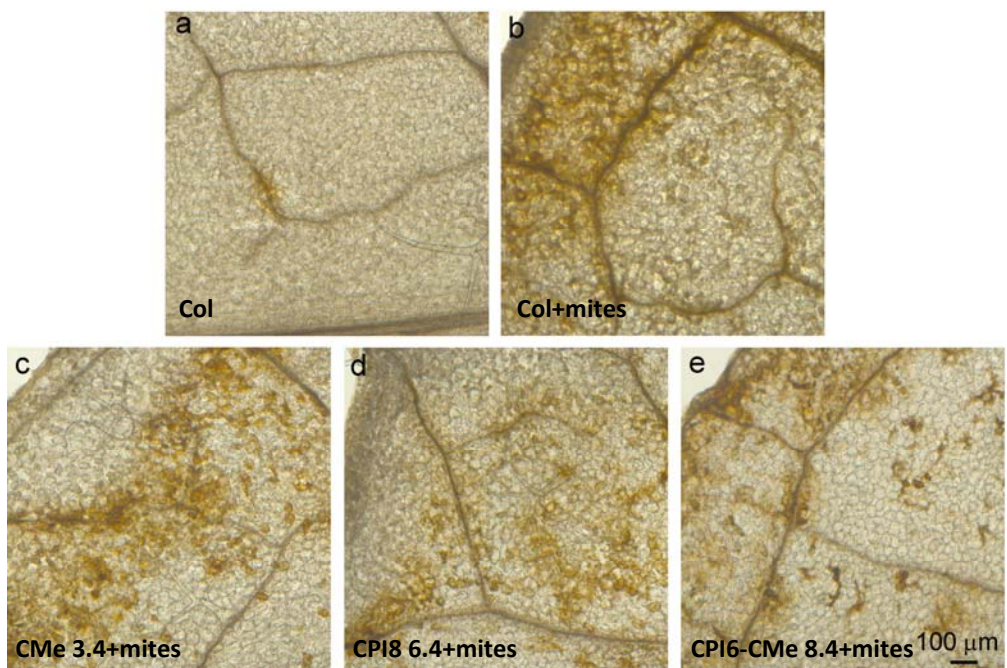

Figure S3

Supplement: Figure S3 — Histochemical detection of reactive oxygen species (ROS) in leaves of control uninfected plants (a) and leaves in response to 24 hours spider mite feeding: b) control Arabidopsis; c) CMe 3.4 line; d) CPI6 6.4 line and e) CPI6-CMe 8.4 line. (PDF) [file pone.0043011.s003.pdf]
