# Supplementary material for: Gene Pyramiding of Peptidase Inhibitors Enhances Plant Resistance to the Spider Mite Tetranychus urticae
Source: PLoS One. 2012 Aug 10;7(8):e43011. doi: 10.1371/journal.pone.0043011 (PMC3416837; doi:10.1371/journal.pone.0043011)
Supplement: Table S1 — Effects of the transgenic Arabidopsis lines on T. urticae development after feeding assay. (PDF) [file pone.0043011.s004.pdf]

Table S1. Effects of the transgenic Arabidopsis lines on *T. urticae* development after feeding assay.

Arabidopsis lines	***T. urticae*** **developing (days) Mean ± SE**
Col	6.7±0.3 [a]
CPI6-CMe 6.4	7.6±0.4 [a]
CPI6-CMe 8.2	8.0±0.5 [ab]
CPI6 6.4	8.4±0.3 [ab]
CPI6 9.8	7.4±0.3 [a]
CMe 3.4	8.4±0.3 [ab]
CMe 8.9	9.7±0.4 [b]

Fifteen newborn larvae mites were placed on detached leaves from Arabidopsis lines and every day the emerged nymphs were counted. Results are expressed as mean ± SE of 6 replicates of (independent plants) of every transgenic line and non-transformed control.

Different letters indicate significant differences ($P<0.05$, Student-Newman-Keuls)
